# The Recovery Spectrum: From Self-Change to Seeking Treatment

**Published:** 2011

**Authors:** Jalie A. Tucker, Cathy A. Simpson

**Keywords:** Alcohol use disorders, alcohol and other drugs recovery, treatment, health care delivery, health service, help-seeking behavior, high-risk groups, screening, brief interventions, guided self-change, telehealth, continuum of care

## Abstract

Recent innovations in alcohol-focused interventions are aimed at closing the gap between population need and the currently uncommon use of alcohol treatment services. Guided by population data showing the heterogeneity of alcohol problems and the occurrence of natural remissions from problem drinking without treatment, alcohol services have begun to expand beyond clinical treatment to offer the untreated majority of individuals with alcohol-related problems accessible, less-intensive services that use the tools of public health practice. These services often are opportunistic, meaning they can be provided in primary-care or other unspecialized health care or community settings. They also can be delivered by nonspecialists, or can be used by people themselves to address problems with alcohol without entering the health care system. This developing spectrum of services includes screening and brief interventions, guided self-change programs, and telehealth options that often are targeted and tailored for high-risk groups (e.g., college drinkers). Other efforts aimed at reducing barriers to care and increasing motivation to seek help have utilized individual, organizational, and public health strategies. Together, these efforts have potential for helping the treatment field reach people who have realized that they have a drinking problem but have not yet experienced the severe negative consequences that may eventually drive them to seek treatment. Although the evidence supporting several innovations in alcohol services is preliminary, some approaches are well established, and collectively they form an emerging continuum of care for alcohol problems aimed at increasing service availability and improving overall impact on population health.

In the past 40 years, the alcohol research field has made great strides in developing evidence-based treatments for alcohol problems (National Institute on Alcohol Abuse and Alcoholism [NIAAA] 2005). Nevertheless, a large gap remains in the United States between treatment need in the population and the small percentage of those who avail themselves of it (Cohen et al. 2005; Substance Abuse and Mental Health Services Administration [SAMHSA] 2007; Wang et al. 2005). Studies suggest that the majority of those with alcohol problems recognize their situation long before they seek treatment, implying that interventions could be provided earlier. Closing the gap between need and service utilization, therefore, is a public health priority that depends on understanding relationships between help-seeking and recovery patterns and processes at both the population and individual levels. This article discusses these issues with an eye towards closing the gap by developing a spectrum of services that matches population need and is sensitive to the preferences of consumers of the services.

Alcohol researchers widely endorse the orienting assumption that the demonstrated heterogeneity in alcohol-related problems, which range from mild to severe, should be served by a system of care that offers a corresponding range of services of varying scope and intensity (Humphreys and Tucker 2002; Institute of Medicine 1990). Although clinical specialty treatment for more serious cases is well established and available, reducing barriers to care and developing appealing, accessible services—particularly for the majority of people with less serious problems—remain priorities. As discussed here, newer intervention options use the tools of public health practice to target risky drinkers and problem drinkers^1^ who delay or avoid seeking specialty alcohol treatment, which often is not needed or appropriate for those with less serious problems. These options collectively comprise a spectrum of “lower threshold” services that can be accessed relatively easily, often without entry into the health care system. These services can be delivered opportunistically when people with alcohol problems are identified in community or nonspecialty medical settings, or individuals with alcohol problems can access them easily and quickly when their motivations shift in favor of sobriety. Examples include screening and brief interventions (SBIs) (e.g., Fleming et al. 2002), guided self-change (GSC) programs (e.g., Sobell et al. 2002), and telehealth options that use phone or computer systems to extend the reach of care (e.g., Hester et al. 2009). A related strategy, also common in public health programs, is to use a “social marketing” approach that targets high- risk groups, or “market segments,” with interventions tailored to address their specific problems and preferences (Walsh et al. 1993). Interventions to reduce binge drinking on college campuses exemplify this approach (e.g., Larimer and Cronce 2007).

To understand the need for a spectrum of services, in this article, we first will summarize population data on alcohol problems and remission rates and describe the U.S. system of care for alcohol-related problems. Next, factors that influence individual decisions to seek care and the outcomes of different pathways for change, with emphasis on self-change, will be discussed. Consideration will then be given to engaging risky and problem drinkers in care by adopting a more consumer-centric approach to service delivery.

## Epidemiology of Alcohol Problems, Help-Seeking, and Remission Patterns

Problems resulting from the use of alcohol are more common than those resulting from the use of all other illicit drugs combined, with 7.5 percent of people aged 12 and older reporting alcohol abuse or dependence during the past year (SAMHSA 2007). Alcohol use peaks during adolescence and early adulthood and then declines over the lifespan (Chen et al. 2004/2005). Males use more alcohol and have more related problems than females at all ages (SAMSHA 2007). Relationships with ethnicity, however, are more complex and variable. For example, recent national survey data showed that Asian Americans and Whites reported the lowest and highest current use of alcohol, respectively (SAMHSA 2006), and Whites were more likely than other ethnic groups to enter treatment.

Despite the high prevalence of alcohol problems, fewer than 25 percent of people with drinking problems seek help from alcohol treatment programs or professionals or from mutual-help groups such as Alcoholics Anonymous (AA) (Cohen et al. 2005; Dawson et al. 2005; Tucker 2003). Alcohol problems and alcohol treatment remain stigmatized, and many who could benefit avoid using services until forced to do so by courts, family members, or employers (Parhar et al. 2008; Schmidt and Weisner 1999). Those who use alcohol-focused services tend to have the most serious problems, particularly comorbid health, mental health, and psychosocial problems (Cohen et al. 2005), and a decade typically elapses between problem onset and recognition and the use of services (Simpson and Tucker 2002). Individuals with drinking problems often surface in other health care venues (e.g., emergency departments or primary care), and untreated alcohol problems add to overall health care costs (Fortney et al. 1999).

Although rates of help-seeking are low in relation to problem prevalence, positive changes in risky alcohol use, including full remissions among people with alcohol use disorders, do occur among those who do not seek care (Dawson et al. 2005; de Bruijn et al. 2006; Tucker 2003). Studies suggest that 66 to 75 percent of resolutions of risky drinking patterns to either abstinence or stable moderation occur naturally without treatment (Klingemann et al. 2001; Sobell et al. 1996). Moreover, the ability to drink in moderation without problems^2^ is a more common outcome among untreated than treated problem drinkers, and population-level data suggest that treatment may shift the relative proportion of good outcomes toward abstinence without necessarily increasing total positive outcomes (i.e., abstinence and moderation combined) (Sobell et al. 1996).

Because treatment seekers generally have more severe alcohol problems, abstinence often is an appropriate drinking goal in treatment programs, whereas moderation is a relatively more common outcome among those who have sought to reduce their drinking naturally and without treatment (Grant 1996). Nevertheless, many who resolve their drinking problems without treatment do meet clinical diagnostic criteria for alcohol use disorders (Dawson et al. 2005), and, conversely, some treatment recipients resume moderate drinking without problems, even if it is not a treatment goal.

Among individuals who receive treatment, positive changes, including abstinence, may occur before, during, or after treatment episodes (Tucker 1995). Cessation of problem drinking may be abrupt or gradual, and treatment appears to help consolidate changes promoted by extratherapeutic factors and to support continued improvement.

Taken together, these findings suggest a more optimistic and variable view of recovery pathways and outcomes than suggested by early clinical studies of treatment seekers, which highlighted the chronic, relapsing nature of alcohol problems and the difficulty of achieving stable abstinence without intensive treatment or lengthy participation in AA (Marlatt and Gordon 1985). Population data instead have shown that, although alcohol problems are prevalent, most affected individuals have less serious problems than the minority who seek treatment and many improve on their own, including achieving stable abstinence or moderation. These findings have raised new research questions about relationships between help-seeking, outcomes of recovery attempts, and drinking problem severity and have spurred the development of a spectrum of services ranging from self-change to specialty treatment. The findings further suggest the need for educating the public regarding the possibility of natural recovery, funding and supporting moderation alternatives, and training mental health professionals to provide moderation services (Sobell and Sobell 2005).

## The Changing U.S. System of Care for Alcohol Use Disorders

Partly in response to population data on alcohol problem prevalence, help-seeking, and recovery patterns, services for alcohol problems have begun to diversify in recent years in ways that are more responsive to the needs and preferences of different population subgroups. Although their availability and use remain limited (SAMSHA 2007), newer professional services include SBIs in nonspecialty settings, GSC, and telehealth options for risky drinkers, problem drinkers, and other high-risk groups such as college students or people living with HIV. Mutual help groups similarly have expanded to include alternatives to AA’s abstinence-oriented 12-step approach (e.g., Rational Recovery, Moderation Management, and Women for Sobriety).

### SBIs

Screening involves brief assessment aimed at the rapid identification of people who are at risk for or have drinking problems. Those so identified either receive brief problem-focused interventions or are referred for specialty alcohol treatment, depending on problem severity. In nonspecialty medical and community settings, SBIs have significant potential to reduce the public health burden of problem drinking through opportunistic intervention. Delivered within minutes in the context of a medical visit or by computer administration while patients wait for care, SBIs have been shown to reduce excessive drinking and other alcohol-related problems and costs (Fleming et al. 2002; Guth et al. 2008; Kypri et al. 2008). Although drinkers with greater problems have been excluded in many studies, controlled trials have shown significant decreases in alcohol consumption and total drinking days for dependent drinkers following SBIs (Brown et al. 2007; Guth et al. 2008), with these drinkers achieving post-SBI effects similar to those of nondependent drinkers. A recent meta-analysis of 21 randomized controlled trials (RCTs) of SBIs found significant decreases in alcohol intake for SBI recipients compared with control participants, although this difference was more pronounced for men than women (Kaner et al. 2007). Each additional minute of intervention added relatively little additional benefit, suggesting that even one-time, minutes-long interventions—as opposed to lengthy ones—are beneficial. Support for the long-term effectiveness of SBIs (more than 12 months), however, has been mixed (Marlatt et al. 1998; Wurzke et al. 2002).

Despite evidence for SBI effectiveness, standardized screening of patients for alcohol problems in primary health care is not routine (Tucker et al., 2010). Expanding brief training on SBI approaches in medical and continuing education is an important need and has been shown to increase screenings by primary care providers (Aspy et al. 2008; Seale et al. 2005).

### GSC

Although less well established than SBIs, community-based GSC programs are another recent innovation aimed at reaching the untreated majority of problem drinkers (e.g., Sobell and Sobell 1993, 2005). GSC programs provide written materials by mail or on Web sites to help drinkers analyze and change their own problem behaviors. Drinkers typically complete brief assessments that allow them to place their drinking practices and problems in a normative context relative to others in the population, set appropriate drinking goals, and guide their change efforts using self-monitoring and behavioral self-control procedures (see Klingemann and Sobell 2007, pp. 244–251 for an international list of self-change Web sites by country).

The research support for GSC programs to date is incipient and somewhat mixed but is generally promising. The approach appears to provide an effective option for risky and problem drinkers for whom clinical treatment may be neither attractive nor feasible. For example, a recent RCT conducted in the Netherlands (Riper et al. 2008) assigned adult heavy drinkers either to a Web-based interactive condition that offered self-paced instruction on cognitive– behavioral and harm-reduction approaches to resolve drinking problems or to a control condition that provided online psychoeducational information about alcohol effects. Reduced drinking to within recommended guidelines was about three times higher in the GSC than in the control condition. Intervention effectiveness at follow-up did not vary by gender, age, or other demographic variables, suggesting broad population utility.

Another RCT (Sobell et al. 2002, 2006) used media advertisements stating that natural recovery was common (e.g., “Did you know that 75% of people change their drinking on their own?”) to recruit 825 untreated problem drinkers who were randomly assigned either to a GSC intervention delivered by mail or to a control condition. GSC participants received personalized drinking feedback and motivational materials based on individual assessments, whereas control participants were mailed alcohol information pamphlets with advice about reducing drinking. Although no significant differences were found between conditions, both groups showed improvements in drinking from baseline levels over a 1-year follow-up period.

These mixed findings raise intriguing questions about how much information or personalization is necessary to facilitate naturally occurring change if it reaches motivated problem drinkers at a “teachable moment.” Subsequent analyses of the GSC study (Sobell and Sobell 2009) concerning when change initially occurred suggested that many drinkers stopped misusing alcohol around the time they saw the media advertisement describing natural recovery as a common pathway to change and decided to call to participate, rather than later in the research when they received either the GSC or alcohol pamphlet materials. Further research on such natural “inflection points” of change can help guide the optimal delivery of opportunistic interventions to facilitate and accelerate the recovery process.

### Telehealth Approaches

Computer-or phone-based telehealth options for problem drinkers have included self-help and social support, health education, and facilitation of treatment entry (Copeland and Martin 2004). Phone-based methods, such as Interactive Voice Response (IVR) assessment, continue to be more accessible than Web-based applications, particularly in poor rural areas, and have been used to monitor drinking and associated risk factors either as a stand-alone supportive intervention or as an aftercare option following treatment (e.g., Mundt et al. 2006; Tucker et al. 2008). Telehealth programs have promise as effective lower-threshold interventions that can be made available for extended intervals outside of the health care system (Humphreys and Tucker 2002), and they may be used to support stepped-care approaches to managing chronic alcohol problems.

Mutual help groups also have been adapted to Internet formats with promising results. For example, Hester and colleagues (2009) randomly assigned heavy drinkers either to a control group in which they received Internet access to Moderation Management resources alone (e.g., self-monitoring, dealing with lapses/relapses) or to an experimental group in which they received access to Moderation Management resources plus a behavioral self-control Web application that they were asked to use at least once a week for 9 weeks. The Web application provided personalized goal setting and feedback on current drinking in relation to goals and had weekly education modules consistent with the Moderation Management approach. Both groups significantly decreased drinking from baseline levels, and improvements were relatively greater for the Moderation Management plus behavioral self-control group. The findings suggest that Internet programs can be used to support moderation and other drinking goals in untreated problem drinkers and that personalized approaches add utility to achieving moderation outcomes. In addition to giving participants knowledge and skills that support behavior change, the online applications allow them to interact with each other over the Web in chat rooms, discussion groups, and the like on an as-needed basis.

### Targeting High-Risk Groups

Several of the preceding approaches have been adapted to target high-risk groups such as youth, individuals at increased HIV risk, or prisoners. Interventions are tailored to the groups’ risks, problems, and contexts. Listings and reviews of evidence-based substance use interventions, including brief interventions and those targeting high-risk and/or underserved groups, may be found at SAMHSA’s National Registry of Evidence-Based Programs (www.nrepp.samhsa.gov) and at the Centers for Disease Control and Prevention’s Diffusion of Effective Behavioral Interventions compendium (www.effectiveinterventions.org).

Many college campuses have moved beyond sanctions-based approaches that have been shown to be largely ineffective in favor of SBI models that can reach large numbers of students and help reduce hazardous (e.g., binge) drinking (Larimer and Cronce 2007), which is a leading cause of morbidity and mortality among young adults (Vogl et al. 2009). Computerized SBI programs (Walters et al. 2005; E-CHUG: http://www.e-chug.com) provide personalized, motivational feedback about risks that are salient to young adults (e.g., risky sexual behaviors or academic consequences) in an interactive approach that may be more effective than conventional didactic formats with young-adult and adolescent drinkers (e.g., Brown et al. 2005).

SBI strategies also show promise as prevention and risk-reduction approaches for drinkers with other health risks. For example, a recent meta-analysis of technology-based HIV prevention interventions, many of which included substance use components, found support for programs that targeted specific groups, used a stages-of-change approach based on individuals’ readiness to change, or allowed for individualized tailoring to participant needs (Noar et al. 2009).

Lastly, treatment often is court ordered for driving under the influence or other drug-related offenses (Schmidt and Weisner 1999). Although the effectiveness of mandated treatment recently has been questioned (Parhar et al. 2008; Sullivan et al. 2008), providing alcohol and drug treatment in prisons improves outcomes after release, as do postrelease aftercare and relapse-prevention programs (Tucker et al. 2010). These programs often make use of public health intervention tools to aid transitions back into the community.

Although opportunistic interventions appear promising in each of these settings, one barrier to assessing their potential is that the full extent to which these services are used in the community is difficult to determine and is not easily assessed in national surveys or research using medical records. For example, SBIs may be delivered in the course of routine health care for medical problems that are not alcohol related, problem drinkers may use GSC programs in the privacy of their homes, and mutual-help groups like Moderation Management and Women for Sobriety typically occur in confidential on-line or real-time group formats. In order to track shifts in utilization as service options diversify, it will be important for research on help-seeking patterns to inquire about the full spectrum of services that fall inside and outside the health care system.

## Influences on Seeking Help for Alcohol Problems

**Table:** Barriers and Incentives to Seeking Help for Alcohol-Related Problems

**General barriers to seeking help:** Social stigma of interventions and asking for help Problem viewed as not serious enough for help Belief that problem can be solved without help
**Treatment-specific barriers:** Concerns about privacy and labeling Lack of insurance/financial resources Waiting lists/inconvenient appointments Pre-entry sobriety requirements/abstinence-only focus Unfavorable opinion of professional treatment
**Common incentives for seeking help from treatment or mutual help groups:** Could not solve problem on own Relationship problems, social encouragement to seek help Occupational/financial problems Knew others who had benefited
**Incentives specific to intervention types:** Want help for alcohol-related health, job, or legal problems (treatment incentive) Convenient meetings at times previously spent drinking (AA incentive)

NOTE: Information summarized from research cited in text.

Although ongoing positive developments exist in the spectrum of alcohol services, their use continues to lag behind population need, and remediating this gap depends on understanding influences on care seeking that operate across individual, social, organizational, and economic levels. The table summarizes research findings concerning barriers to and incentives for seeking help for alcohol problems. Some influences apply to all forms of help-seeking, whereas others pertain to specific services (e.g., formal treatment). Foremost among the general barriers is the enduring stigma associated with alcohol problems and specialty treatment (Saunders et al. 2006). People with alcohol problems understandably are reluctant to seek help until forced to do so or until their problems become severe (Calderia et al. 2009; Cunningham et al. 1993; Storbjork and Room 2008).

Reluctance to seek help typically is not attributed to denial. Problem recognition occurs early in problem development, but seeking help is a late event, if it occurs at all (Simpson and Tucker 2002). Drinking problem onset typically occurs in the early to mid-20s age-group, whereas patients in speciality treatment often are in their early to mid-40s (Tucker et al. 2010). During the intervening years, the majority of people with problems “mature out” without interventions, some surface in the health care system and use costly medical services that do not address their drinking problems, and the small minority that finds its way into specialty treatment tends to have more severe alcohol dependence and greater alcohol-related health, legal, and other life problems (Cohen et al. 2007; Cunningham and Breslin 2004; Tucker 2003).

The functional consequences of drinking, particularly in the social arena—and not heavy drinking, per se—drive help-seeking (Tucker 2003; Weisner et al. 2003). Negative social and health consequences are particularly strong predictors of treatment entry (Hajema et al. 1999). Absent such forces, many problem drinkers report that their problems are not severe enough for treatment, and they seek help from family and friends, clergy, or mutual-help groups (Narrow et al. 1993; Wild et al. 2002). Decisions to seek formal help or to handle the problem without professional assistance also depend on how favorably or unfavorably potential consumers view such professional resources (Cellucci et al. 2006).

Help-seeking and positive behavior change are more likely if an individual’s social network encourages help-seeking and discourages heavy drinking (Codd and Cohen 2003; George and Tucker 1996; Longabaugh et al. 2001), and the converse also is true. Such findings have guided interventions that involve the social network. The Community Reinforcement and Family Therapy (CRAFT) approach, described in the sidebar, is designed to promote treatment entry through incorporation of the concerned friends and family members of treatment-resistant drinkers (Smith and Myers 2004). Alcohol treatment systems also have functional and logistic deficiencies that have the potential for improvement in order to facilitate help-seeking (Humphreys and Tucker 2002). Specialty alcohol treatment programs and mutual-help groups like AA emphasize lifelong abstinence, which may be appropriate for people with serious problems but is unappealing and unnecessary for those with milder problems. Treatment programs, especially in the public sector, often have long waiting lists and inconvenient scheduling. Limited service availability, treatment cost, and inadequate insurance coverage can impose additional barriers (Saunders et al. 2006; Tucker et al. 2004). Until the passage of Federal parity legislation in 2008, alcohol and drug treatment services typically had more limited insurance coverage (often with low lifetime limits) compared with comparable medical problems. The U.S. Patient Protection and Affordable Care Act of 2010 reaffirmed parity requirements.

Greater worry about the cost of treatment has been observed among nontreatment seekers compared with those engaged in services (Saunders et al. 2006). Such functional and financial concerns appear to be of greater significance for problem drinkers who are female, minority, or lower income (Saunders et al. 2006; Snowden 2001). Although mutual-help groups like AA are widely available alternatives, these groups are not universally appealing, and low-threshold professional options are not yet widely available.

## Increasing the Appeal of Services to Consumers of Care

The treatment field has made considerable progress over the past decade in bringing alcohol services into routine health care. Incorporation of SBIs into primary-care and other nonspecialty medical settings has been especially beneficial. These brief opportunistic interventions increase case finding and access to care, while helping to reduce the associated stigma of alcohol treatment (Tucker et al. 2010).

Beyond improvements in access, however, the appeal of alcohol services could be increased by expanding treatment goals beyond the usual focus on eliminating alcohol use. Many treatment programs emphasize immediate abstinence and often require it prior to treatment entry, but research on resolution patterns has revealed several pathways to successful change (Sobell et al. 1996; Tucker 2003). For example, goal setting to reduce drinking levels can involve a continuum of behavioral interventions and gradual approaches to attaining healthier behavior patterns instead of requiring abstinence in the near term. The appeal of services also can be enhanced by addressing the functional problems and risks related to problem alcohol use that often motivate treatment entry (e.g., problems with intimate, family, or social relationships; health or legal concerns).

Another approach involves making changes in the accessibility, organization, and financing of systems of care in ways that enhance treatment entry and engagement. Examples of service features that have been found important or attractive to alcohol and other drug misusers and their families include convenient appointments, parking, and childcare; availability of HIV testing and other substance-relevant services; and lower cost and insurance coverage (Tucker et al. 2009). As discussed in the sidebar, providing “treatment on demand” by offering same- or next-day appointments (Kaplan and Johri 2000; Sorensen et al. 2007) capitalizes in a positive way on the changing motivations of substance users between sobriety and continued use.

More generally, treatment providers are beginning to view problem drinkers, illicit drug users, and their social networks as consumers of services who exercise choice among many available alternatives, including seeking help or continuing substance use (e.g., Fountain et al. 2000; Tucker et al. 2009). This concept highlights the need to make services more user friendly and attractive to the consumer base in order to increase the reach and impact of a spectrum of alcohol services on population health. The preferences of consumers, however, are not always in line with professional views of therapeutic or appealing attributes, as represented in evaluation studies of treatment efficacy (Seligman 1995). Professionals tend to focus on technical variations between different treatments, whereas consumers of services tend to focus on and value non-technical dimensions. For example, a recent State-level survey assessed consumer preferences for professional, mutual-help, and lay helping resources, as well as more anonymous computerized and self-help resources (Tucker et al. 2009). Respondents preferred help that involved personal contact compared with computerized help or self-help, but they were indifferent whether personalized help was dispensed by professional or lay providers. Respondents in households with a member who had alcohol or drug problems rated services more negatively, especially if services had been used.

In conclusion, viewing people with alcohol problems and their social networks as consumers directs attention to the need for improving and marketing a system of care that is consumer focused. This will require greater understanding of population need, tailoring interventions to population subgroups and individual circumstances, and formulation of substance-related goals that are consistent with the current situations, beliefs, and preferences of subgroups and individuals. Future research should address these issues and seek to guide the development of accessible pathways to different types and levels of care. Future studies also will benefit by including of drinkers across the spectrum of potential alcohol problems (from mild to severe) and moving beyond the longstanding focus on treatment-seeking samples. Selecting from among a range of interventions should be a mutual, consumer-informed process, and the growing availability of a range of services to support recovery is a welcome development after decades of a “one size fits all” treatment system that served only a small segment of those who might be open to receiving help for their problems.

**Facilitating Help-Seeking Among Problem Drinkers**

Just as there are both clinical and public health approaches to intervention, a duality exists among approaches to promoting help-seeking, including increasing individuals’ motivation to seek help and creating contexts to increase the likelihood that problem drinkers will use available services. One approach is epitomized by the longstanding view held by Alcoholics Anonymous (AA) that alcoholics are in denial until they “hit bottom” and recognize their problem, as evidenced by receptiveness to seek help. The concept of denial has been empirically challenged by findings showing that problem drinkers generally recognize their problem but delay or forego treatment seeking because of the associated stigma, unfavorable prior treatment experiences, privacy concerns, inflexible abstinence goals, and the like (Cunningham et al. 1993; Simpson and Tucker 2002).

A second approach to engaging reluctant substance users in treatment alters both personal and contextual factors relevant to seeking help. The Community Reinforcement and Family Training (CRAFT) model (see Smith and Myers 2004) has its roots in the Community Reinforcement Approach (CRA; Hunt and Azrin 1973) and related contingency management approaches to alcohol and drug treatment (Higgins et al. 1991; Smith et al. 2001). In these models, alcohol problems are viewed largely as disorders of reinforcement that are directly amenable to positive changes through shifts in an individual’s ongoing environmental context that make a non–drug-using lifestyle more rewarding than one focused on substance use. CRA seeks to rearrange the substance misuser’s environment through cognitive–behavioral approaches that focus on the involvement of social networks; individualized analysis of triggers, rewards, and consequences of substance use; and behavioral skills training.

The CRAFT model is an extension of CRA and focuses on concerned significant others (CSOs), rather than on treatment-resistant substance misusers themselves (Smith et al. 2001; Waldron et al. 2007). CRAFT leverages the already-significant role that CSOs have been shown to play in promoting treatment entry (e.g., Miller et al. 1999) by teaching CSOs behavior management techniques to modify their interactions with the drug-using individual, as well as cognitive–behavioral strategies to reduce enabling behaviors and communication strategies to increase receptivity to treatment (Smith and Myers 2004). CRAFT has shown some success in engaging treatment resistant illicit substance users. Across studies, rates of treatment engagement with CRAFT have been approximately twice that of AA and other family approaches (Waldron et al. 2007). More work is needed with respect to engagement in treatment for alcohol use disorders specifically.

A third approach exemplified by “treatment on demand” initiatives (Carr et al. 2008) does not seek to change individual motivation directly. Instead, this approach creates a context of choice that promotes rapid treatment entry when individuals’ shifting motivations for drug use versus sobriety change in favor of the latter. This opportunistic approach makes it easy for problem drinkers and illicit drug users to make “impulsive” choices to seek care by minimizing the delay to receipt of services (i.e., same- or next- day appointments). People with addictive disorders generally show faster devaluation or “discounting” of delayed rewards compared with people without such problems (Bickel and Marsch 2001). The greater the discounting, the more likely treatment-on-demand programs are to help impulsive problem drinkers enter care. This is an example of a policy approach known as asymmetric paternalism (Loewenstein et al. 2007), which creates choice situations that selectively promote beneficial choices among persons with biased decision-making styles (e.g., steep discounting), without unduly limiting freedom of choice among those with less biased styles. Evidence does not suggest that rapid treatment entry results in greater attrition (Tucker and Davison 2000), although greater baseline impulsivity on discounting and related choice tasks have been associated with poorer short-term treatment outcomes (e.g., MacKillop and Murphy 2007;; Yoon et al. 2007). Thus, offering treatment on demand appears to help to attract selectively those problem drinkers who are more impulsive and more likely to need extensive services to recover.

—Jalie A. Tucker and Cathy A. Simpson

## Footnotes

1 This article adopts the common assumption that drinking practices and problems lie along a continuum from abstinence to high-risk use and follow the NIAAA (2005) decision tree for making initial decisions about problem severity. The interventions discussed generally are targeting people identified as having mild to moderate problems that fulfill clinical criteria for an alcohol use disorder (typically alcohol abuse) or who are engaging in risky drinking patterns that may fall short of formal clinical diagnosis. The terms “problem drinker” and “risky drinker” are used to refer to appropriate recipients of these interventions without specific regard to formal diagnostic status, which often is unknown or only partially established outside of clinical settings in the venues in which these services typically are used.

2 Criteria for safe versus hazardous drinking vary somewhat from country to country (International Center for Alcohol Policies, 2003; NIAAA, 2005; World Health Organization 2000). Several systems place thresholds for hazardous drinking at >5 drinks/day for men and >4 drinks/day for women. Recommended patterns for low-risk drinking typically are set at <3 drinks/day with <21 drinks/week for men and <2 drinks/day with <14 drinks/week for women. The NIAAA (2005) recommends somewhat lower levels. In addition to low-risk drinking practices, successful moderation involves an absence of alcohol-related problems (e.g., health, psychosocial).
